# YOLO-SAM AgriScan: A Unified Framework for Ripe Strawberry Detection and Segmentation with Few-Shot and Zero-Shot Learning

**DOI:** 10.3390/s25247678

**Published:** 2025-12-18

**Authors:** Partho Ghose, Al Bashir, Yibin Wang, Cristian Bua, Azlan Zahid

**Affiliations:** 1 Department of Biological and Agricultural Engineering, Texas A&M AgriLife Research, Texas A&M University System, Dallas, TX 75252, USA; partho.ghose@tamu.edu (P.G.); albashir@tamu.edu (A.B.); yibinwang@tamu.edu (Y.W.); cristian.bua@phd.unipi.it (C.B.); 2 Department of Information Engineering, University of Pisa, 56122 Pisa, Italy

**Keywords:** precision agriculture, strawberries, few-shot, detection, YOLO, SAM, zero-shot, segmentation

## Abstract

Traditional segmentation methods are slow and rely on manual annotations, which are labor-intensive. To address these limitations, we propose YOLO-SAM AgriScan, a unified framework that combines the fast object detection capabilities of YOLOv11 with the zero-shot segmentation power of the Segment Anything Model 2 (SAM2). Our approach adopts a hybrid paradigm for on-plant ripe strawberry segmentation, wherein YOLOv11 is fine-tuned using a few-shot learning strategy with minimal annotated samples, and SAM2 performs mask generation without additional supervision. This architecture eliminates the bottleneck of pixel-wise manual annotation and enables the scalable and efficient segmentation of strawberries in both controlled and natural farm environments. Experimental evaluations on two datasets, a custom-collected dataset and a publicly available benchmark, demonstrate strong detection and segmentation performance in both full-data and data-constrained scenarios. The proposed framework achieved a mean Dice score of 0.95 and an IoU of 0.93 on our collected dataset and maintained competitive performance on public data (Dice: 0.95, IoU: 0.92), demonstrating its robustness, generalizability, and practical relevance in real-world agricultural settings. Our results highlight the potential of combining few-shot detection and zero-shot segmentation to accelerate the development of annotation-light, intelligent phenotyping systems.

## 1. Introduction

Strawberry is one of the most economically valuable and widely consumed fruits in the United States, with an annual production of 1.33 million tons in 2021, valued at 3.42 billion [1]. Meeting this demand is increasingly challenging because of environmental constraints and a shortage of labor. Hence, precision agriculture, particularly the advancement of artificial intelligence (AI), has emerged as a vital strategy to address these challenges, offering advanced technological solutions that improve fruit management, optimize yields, and promote sustainable farming practices [2]. Computer vision (CV) plays an important role in this paradigm. By allowing the accurate detection and segmentation of fruits, such as strawberries, CV facilitates key agricultural applications, including automated harvesting, yield estimation, and plant health monitoring [3], and thus helps the grower to reduce labor costs significantly.

In recent decades, many studies have highlighted the relevance of the application of the CV approach for strawberry fruit detection and segmentation. For example, Bashir et al. [4] developed a YOLOv8-based CV system to detect the four stages of strawberry growth from flowering to ripening and count them in real time. He et al. [5] developed YOLOv5s-Straw, a customized YOLOv5-based detector, which achieved an mAP of 80.3% across three maturity stages, although it lacked fine-grained segmentation capabilities. Recently, Crespo et al. [6] proposed an efficient Mask R-CNN pipeline optimized with TensorRT to achieve both high segmentation accuracy and real-time processing speed. Moreover, StrawSnake, introduced by Guo et al. [7] based on a contour learning approach, produced an mIoU of 81.54%, emphasizing its ability to segment complex boundaries, albeit under supervised learning constraints. Despite their advantages, conventional CV approaches in agriculture are hindered by their heavy reliance on manual annotation. Segmentation requires domain experts to delineate object boundaries at the pixel level. This process is not only time-consuming but also labor-intensive, as it requires technical expertise and effort to ensure accuracy [8,9]. This challenge highlights the necessity for methods that can reduce the reliance on manual labeling while ensuring high performance.

Zero-shot learning (ZSL) has emerged as a promising solution for reducing data dependency in agricultural CV tasks [10]. ZSL enables models to generalize to unseen categories without explicit training on the target dataset by leveraging semantic relationships, textual descriptions, and attribute embeddings [11,12,13]. Foundation models, particularly the Segment Anything Model 2 (SAM2) [14], have shown excellent performance for segmentation in ZSL settings and are capable of generating pixel-wise boundaries of objects across diverse image domains. However, SAM2 relies heavily on manual expert prompts to achieve class-specific segmentation [14]. This dependence on human input restricts its automation and scalability, particularly when dealing with large-scale agricultural datasets. To mitigate this limitation, a self-prompting strategy can be employed, in which prompts are generated automatically to guide SAM2 toward object-specific segmentation rather than generic object delineation. Integrating an object detection model, such as YOLO, can generate prompts for the SAM2 model to facilitate object-specific segmentation rather than segmenting all objects from the images.

Recent studies have demonstrated the effectiveness of YOLO-SAM integration for agricultural applications. For instance, Huang et al. [15] proposed a framework for strawberry canopy segmentation by integrating YOLO8 to guide SAM to produce precise segmentation masks, obtaining a mean intersection over union (mIoU) of 0.749. A plant leaf recognition system was introduced by Zhao et al. [16], following a similar strategy YOLO8 + SAM), for three different plant species (lilac, field cotton, and mulberry-leaf peony) and attained a detection accuracy of 87% with an inference time of 0.03 s per image. Reddy et al. [17] applied YOLO7 and YOLO8 with SAM on images captured by unmanned aerial vehicles (UAVs), and their experimental findings demonstrated $R^{2}$ of 0.913 for cotton yield prediction. Moreover, a similar approach was adopted by [18] for mango orchard canopy delineation, applying YOLO7 as a detection model with SAM. These studies consistently reported improved segmentation accuracy, annotation efficiency, and yield estimation reliability. Furthermore, methods such as YOLO–DINO–SAM in panoptic segmentation tasks (PhenoBench dataset) for weeds achieved high PQ+ scores (≈81) [19], demonstrating the potential of combining detection and foundation models for hierarchical agricultural scene understanding. Although the above-mentioned studies underscore the efficiency of different YOLO versions for object detection, the standard YOLO framework is not inherently zero-shot or few-shot and requires annotated datasets for training. To extend the YOLO capabilities toward ZSL, variants such as YOLO-World were introduced, which incorporate text embeddings and open-vocabulary detection to recognize unseen classes [20]. However, within our agricultural context, ZSL-based YOLO models exhibit limited performance because of the substantial visual complexity, pervasive occlusions, and the fine-grained distinctions among plant organs, especially in strawberry plants. Few-shot learning (FSL) can provide a practical alternative to address these challenges. FSL fine-tunes pretrained models using a limited number of labeled data, typically ranging from a handful to a few hundred [21,22]. By leveraging FSL, the dependence on extensive manual annotations can be substantially reduced, thereby facilitating prompt generation and adaptation in ZSL scenarios.

To address this gap, we propose YOLO-SAM AgriScan, combining ZSL and FSL paradigms together, integrating YOLOv11-based few-shot detection with SAM2-based zero-shot segmentation. YOLOv11 was fine-tuned under the FSL paradigm to detect ripe strawberries by providing bounding-box (Bbox) prompts, which were subsequently passed to SAM2 for high-resolution segmentation. This unified design effectively merges the strengths of both paradigms, leveraging FSL’s adaptability to limited data and ZSL’s generalization ability, to achieve precise, scalable, and annotation-efficient strawberry segmentation.

The key contributions of this study are as follows:

1. An FSL framework for ripe strawberry detection using YOLOv11 is established, trained with varying image counts (50, 100, and 200) and epoch numbers (5, 10, and 20) to evaluate performance under limited data conditions.
2. A self-prompting mechanism is introduced to integrate the trained YOLOv11 with SAM2, enabling ZSL for segmentation. This approach eliminates the need for additional pixel-level annotations and postprocessing by guiding SAM2 to perform object-specific segmentation based on YOLO-detected Bboxes.

The remainder of this paper is organized as follows: Section 2 details the methodology, covering data acquisition, preprocessing, and model development. The detailed experimental setup is described in Section 2.5. Section 3 presents the experimental results of the proposed method. Finally, Section 4 concludes the paper and outlines directions for future work.

## 2. Methodology

### 2.1. Data Acquisition and Processing

To ensure wide applicability, the proposed model was trained and validated on two distinct datasets: our own greenhouse-grown strawberry dataset ($D_{1}$) and a publicly available field-grown strawberry dataset ($D_{2}$).

**Figure 1:**
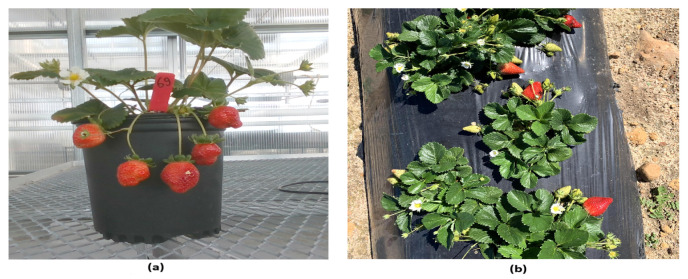
Sample images from datasets: (**a**) an image of a greenhouse-grown strawberry plant with some hanging fruits and (**b**) an image of field-grown strawberry plants with some fruits lying on the soil bed.

- **Greenhouse-grown strawberry data ($D_{1}$):** This dataset consists of 300 images of greenhouse-grown strawberry plants, each with a resolution of 1280 × 720 pixels. Greenhouse-grown strawberries typically hang from the plant without contacting the soil, as illustrated in Figure 1a. We potted 114 strawberry plants in standard 1-gallon (3.78 L) nursery pots and cultivated them on tabletops in a greenhouse at the Texas A&M AgriLife Research and Extension Center in Dallas, TX. An Intel^®^ RealSense™ D435i camera (Intel Corporation, Santa Clara, CA, USA) was used to collect data, targeting on-plant ripe strawberries. Images captured under low-light conditions were enhanced by increasing the brightness by 20% and applying gamma correction (γ=0.7) to improve the visual quality.
- **Field-grown strawberry data ($D_{2}$):** We also adopted a field-grown strawberry dataset collected from Roboflow universe [23], where strawberry plants and fruits often lie directly on the soil bed, as shown in Figure 1b. This dataset contains 900 images with a resolution of 3024 × 4032 pixels, similarly targeting ripe strawberries like dataset $D_{1}$.

Dataset $D_{1}$ was annotated using CVAT (CVAT.ai Corporation, Wilmington, DE, USA) [24]. Bbox was used for each ripe strawberry and labeled as ‘ripe’. In addition, we outlined the polygon of the corresponding strawberries to evaluate the segmentation performance of the proposed zero-shot segmentation model. On the other hand, we utilized the annotated label files of dataset $D_{2}$, where ripe strawberries were already labeled as ‘strawberry’.

### 2.2. Fine-Tuning YOLOv11 for Detection

YOLOv11 was fine-tuned and trained with few-shots for obtaining the prompt Bbox of ripe strawberry. The architecture of YOLOv11 integrates three key components: the C3K2 block, SPPF module, and C2PSA block [4]. The C3K2 block applies lightweight 3×3 convolutions to reduce the number of parameters while retaining high-quality feature extraction. For an image *i* with *n* candidate regions, the detection process can be formulated as

(1) $${\hat{Y}}_{i}={\left\{\left(b_{i,j},c_{i,j},p_{i,j}\right)\right\}}_{j=1}^{n}$$

where $b_{i,j}$ denotes the Bbox parameters, $c_{i,j}$ the predicted class label, and $p_{i,j}$ the confidence score for instance *j*.

**Figure 2:**
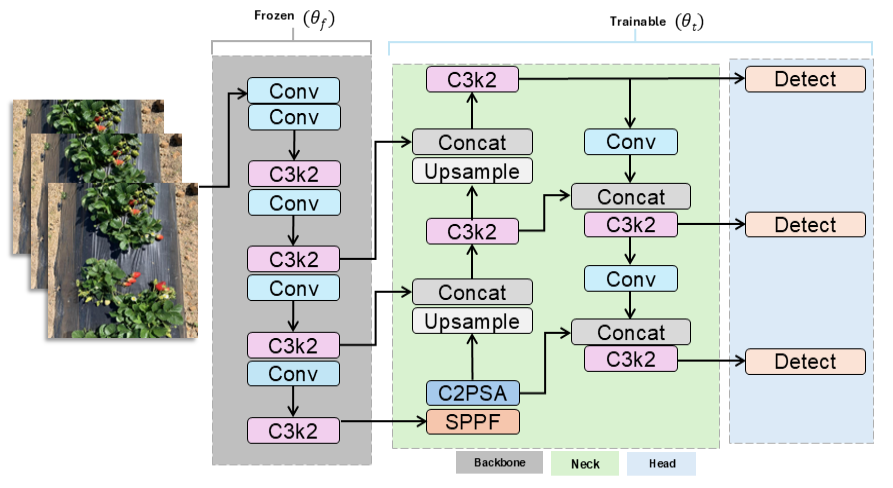
Detailed architecture of YOLOv11 model.

Transfer learning was also carried out for the pretrained model, as illustrated in Figure 2. The backbone layers were frozen to preserve previously learned low-level features, such as edges, textures, and shapes, whereas the neck and detection head were fine-tuned using strawberry images.

This approach reduces the training cost, stabilizes the convergence, and prevents overfitting on a limited dataset. Formally, given the model parameters $\theta =\{\theta_{f},\theta_{t}\}$, where $\theta_{f}$ is the frozen backbone weights and $\theta_{t}$ is the trainable neck and head weights, the training updates are applied on $\theta_{t}$, carried out by the below equation:

(2) $$\theta_{t}\leftarrow \theta_{t}-\eta \;\nabla_{\theta_{t}}L\left(\hat{Y},Y\right)$$

where η is the learning rate, and L is the detection loss that combines the classification, localization, and objectness terms. The gradient $\nabla_{\theta_{t}}L$ indicates how the loss changes with respect to the trainable parameters, guiding the optimization process during fine-tuning. This ensured that YOLOv11 leveraged pretrained general features while adapting effectively to strawberry-specific detection.

### 2.3. SAM2 for Segmentation

SAM2 is a segmentation framework developed by Meta. It introduces a unified architecture that combines image and video segmentation capabilities, thereby enabling seamless performance across diverse visual data. In this work, only the image segmentation pathway of SAM2 is used, as our dataset consists entirely of single images. The model leverages large-scale pretraining over 600,000 annotations, allowing it to generalize its zero-shot segmentation capabilities. It can generate segmentation masks based on Bboxes, key points, or textual descriptions. The key components of SAM2 are the (i) image encoder, (ii) prompt encoder, (iii) memory mechanism, and (iv) mask decoder.

The image encoder is built on a transformer backbone and is responsible for extracting rich visual features from data. It interprets the scene at each time step, forming the foundation of the segmentation pipeline. The prompt encoder is designated for user instructions or prompts, guiding the model to focus on particular objects or regions of interest (RoIs). The memory mechanism, combined with a memory encoder, a memory bank, and an attention mechanism, is responsible for temporal consistency. This design allows the model to recall the information from the previous frames. The mask decoder generates the segmentation masks by combining visual features and user prompts, producing accurate delineations of the RoI. Although SAM2 incorporates a memory module for temporal consistency in video segmentation, this component was not utilized in our framework.

### 2.4. Proposed Hybrid Framework

To obtain pixel-accurate instance masks of ripe strawberries as the target RoI in the FSL and ZSL paradigms, a two-stage pipeline combining a tuned YOLOv11 detector with SAM2 was proposed (Algorithm 1). In this framework, YOLOv11 is responsible for generating the Bbox prompt of the target, and SAM2 extracts the fine-grained mask of the instance inside the Bbox.

Let $I=\{I_{1},\ldots ,I_{N}\}$ be the input images. For each $I_{k}$, YOLOv11 produces candidate Bboxes ${\hat{B}}_{k}$ (Equation (3)), which are used as prompts for SAM2. SAM2 fuses image features with prompt features and decodes the final segmentation mask, denoted as ${\hat{M}}_{k}$ (Equation (4)).

(3) $${\hat{B}}_{k}=f_{\theta }\left(I_{k}\right)$$

(4) $${\hat{M}}_{k}=g_{\phi }\;\left(I_{k},\;{\hat{B}}_{k}\right)$$

where $f_{\theta }$ denotes the YOLOv11 detector with parameters θ, and $g_{\phi }$ denotes the SAM2 segmenter with parameters ϕ.
**Algorithm 1:** YOLO-SAM AgriScan: Bounding-box-prompted ripe strawberry segmentation**Input**: Input image set $I=\{I_{1},I_{2},\ldots ,I_{N}\}$**Output**: Segmentation masks $M=\{M_{1},M_{2},\ldots ,M_{N}\}$  1**Step 1: Detection**  2  Freeze backbone layers $\theta_{f}$, train detection head $\theta_{t}$  3  For each image $I_{k}\in I$:  4    Predict Bboxes ${\hat{B}}_{k}=f_{\theta }\left(I_{k}\right)$  5    where ${\hat{B}}_{k}=\left\{b_{k,1},b_{k,2},\ldots ,b_{k,m}\right\}$  6**Step 2: Segmentation**  7  Encode image: $E_{k}=\phi_{img}\left(I_{k}\right)$  8  Encode prompts (Bboxes): $P_{k}=\phi_{prompt}\left({\hat{B}}_{k}\right)$  9  Decode segmentation masks: ${\hat{M}}_{k}=\phi_{mask}\left(E_{k},P_{k}\right)$10**Output**11  Return $M=\{{\hat{M}}_{1},{\hat{M}}_{2},\ldots ,{\hat{M}}_{N}\}$

In Step 1 of the algorithm, transfer learning was adopted by freezing the low-level backbone parameters $\theta_{f}$ and training only the task-specific layers $\theta_{t}$ (neck + detection head) on the dataset, as depicted in Equation (5).

(5) $$\theta =\theta_{f}\cup \theta_{t},\;\theta_{f}\;\;\mathrm{frozen},\;\theta_{t}\;\;\mathrm{trainable}.$$

Given $I_{k}$, YOLOv11 predicts a set of *m* Bboxes, ${\hat{B}}_{k}={\left\{b_{k,i}\right\}}_{i=1}^{m}$, where $b_{k,i}=(x_{k,i},y_{k,i},$$w_{k,i},h_{k,i},s_{k,i},c_{k,i})$. Here, (x,y,w,h) are the box parameters, *s* is the confidence score, and *c* is the class (ripe). Non-maximum suppression (NMS) was applied to remove redundant detections. The detector was trained using standard YOLO losses.

**Figure 3:**
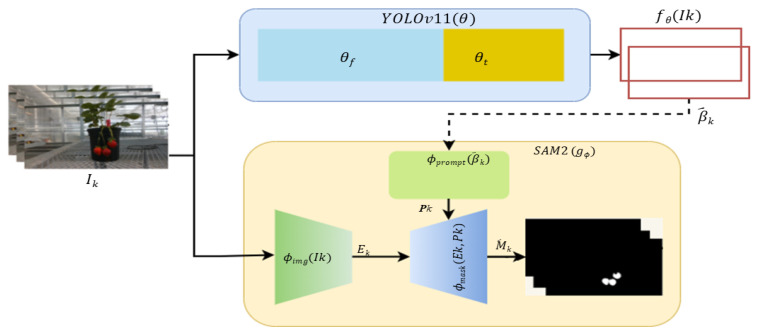
Schematic diagram of YOLO-SAM AgriScan framework for ripe strawberry segmentation.

In Step 2, SAM2 receives the image $I_{k}$ and the bounding boxes ${\hat{B}}_{k}$ as prompts. The image encoder first computes a dense embedding $E_{k}=\phi_{\mathrm{img}}\left(I_{k}\right)$. The prompt encoder $\phi_{\mathrm{prompt}}\left({\hat{B}}_{k}\right)$ then maps each bounding-box prompt into a corresponding embedding $P_{k}$. Because our work utilizes only single images and does not involve video sequences, SAM2’s memory mechanism was not used. Therefore, no recurrent memory state is propagated, and no temporal fusion was applied. The mask decoder directly combines the image embedding $E_{k}$ with the prompt embedding $P_{k}$ to generate the pixel-level segmentation mask ${\hat{M}}_{k}$. The final output is a set of masks $M=\{{\hat{M}}_{1},\ldots ,{\hat{M}}_{N}\}$, each delineating ripe strawberries at the pixel level. A schematic of the proposed model is shown in Figure 3.

### 2.5. Model Training

We selected YOLOv11m and fine-tuned it for adopting FSL on both $D_{1}$ and $D_{2}$ datasets. The input image size was set to 640 × 640 pixels, and the batch size was eight. An extensive data augmentation technique was utilized during training, such as color shifting, rotation, scaling, and translation, to adapt the model to the real-world conditions. AdamW was used as an optimizer while the initial learning rate was set at 0.0001 with a decay factor of 0.00001. A weight decay of 0.0005 was applied for the regularization. To preserve the pretrained low-level features, the first four backbone layers were kept frozen, whereas the detection head remained trainable to adjust to the characteristics of the strawberry dataset. Early stopping was implemented to prevent the model from overfitting. The training was conducted in a CUDA-enabled $Intel^{\left(R\right)}$$Core^{\left(TM\right)}$ Ultra 7 165H processor (1.40 GHz) PC, equipped with 32 GB RAM, and an NVIDIA RTX A500 GPU with 4 GB VRAM.

In the first scenario, we used 80% of the dataset for training and evaluated the effect of different training durations by running the model for 5, 10, and 20 epochs, respectively. This allowed us to examine how the training length influences the detection performance. In the second scenario, we applied an FSL setting by training the detector with subsets of 50, 100, and 200 images randomly sampled from the original training split. This setup was designed to assess the model performance under limited annotated data. In both scenarios, 20% of the data was consistently used as the test set. Based on these experiments, the best-performing detection configuration in term of images used and epochs—training with 50 images for 10 epochs—was selected as the detection backbone for the subsequent ZSL stage for both the $D_{1}$ and $D_{2}$ datasets as details in Table 1.

### 2.6. Performance Evaluation Matrices

Standard metrics were used to assess the proposed methodology for detection and segmentation. For detection, we employed standard precision (P) and recall (R) metrics. We also use mean average precision (mAP), the standard detection metric that computes the average precision across classes and IoU thresholds, reflecting both localization accuracy and correct class prediction.

The PR curve plots the precision against the recall at different detection thresholds, providing a visual representation of the model performance. A larger area under the PR curve (AUC-PR) indicates better detection capability.

As the segmentation approach follows a *zero-shot* paradigm, we evaluated the segmentation performance on the test data only using Equations (6) and (7).

(6) $$DSC=\frac{2\times |A\cap B|}{\left|A|+|B\right|}$$

(7) $$IoU=\frac{\left|A\cap B\right|}{\left|A\cup B\right|}$$

where *A* represents the predicted mask, and *B* represents the ground-truth mask.

## 3. Results and Discussion

### 3.1. Detection Performance

#### 3.1.1. Performance at Different Epochs

**Figure 4:**
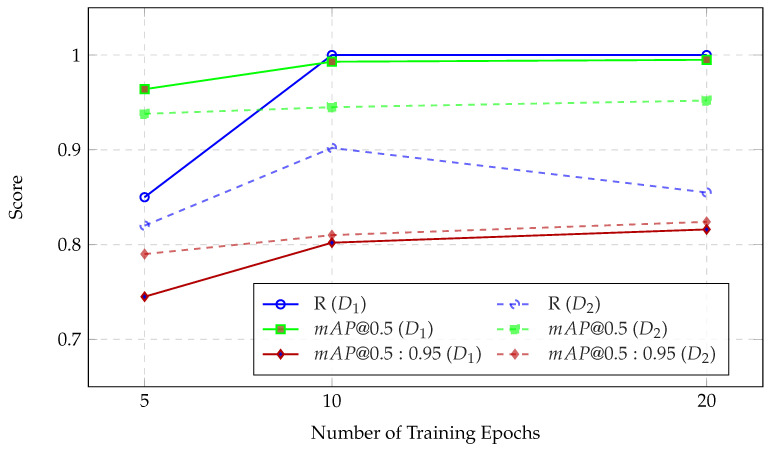
Effect of training epochs on detection performance across datasets $D_{1}$ and $D_{2}$.

Figure 4 displays performance across different training epochs. The analysis focuses on three metrics: recall (*R*), mean average precision (mAP) at an IoU of 0.5 (mAP@0.5), and mAP over IoU ranging from 0.5 to 0.95 (mAP@0.5:0.95).

**Figure 5:**
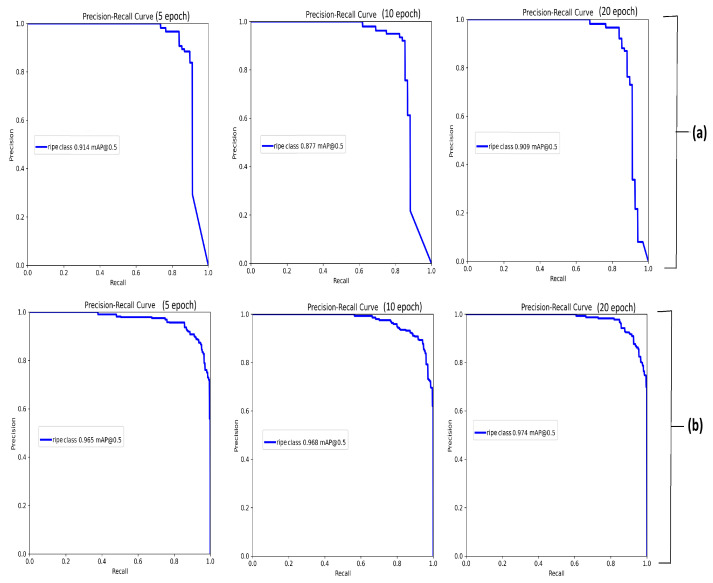
PR curves for ripe strawberry detection for different epoch numbers (**a**) for $D_{1}$ and (**b**) $D_{2}$ datasets.

For $D_{1}$, the model demonstrates a consistent improvement when the number of training epochs increases. At 5 epochs, the *R* is measured at 0.85, with an mAP@0.5:0.95 of 0.745. By 10 epochs, *R* reached a perfect score of 1.0, indicating that correspondingly, mAP@0.5 improved substantially to 99.3%, reflecting accurate object localization. At 20 epochs, the model exhibited signs of performance saturation, with mAP@0.5 plateauing at 0.995. However, mAP@0.5:0.95 increased further to 0.816, suggesting enhanced precision in Bbox localization across stricter IoU thresholds. In contrast, the model’s performance on the $D_{2}$ dataset shows a more nuanced trend. Recall initially increases from 0.853 at 5 epochs to 0.902 at 10 epochs but declines to 0.855 at 20 epochs. This decline may indicate overfitting or sensitivity to annotation variability and noise within the dataset. Despite this, mAP@0.5 exhibited a steady upward trend, increasing from 0.942 to 0.952 across the same epoch range, suggesting the continued learning of general object localization patterns. The mAP@0.5:0.95 metric improved more modestly, from 0.80 to 0.824, reflecting incremental gains in fine-grained localization. In addition, the performance was evaluated using PR curves and mAP@0.5 for different epochs, as shown in Figure 5. It is evident from the metrics that the model performs slightly better on $D_{1}$ than on $D_{2}$, likely due to $D_{1}$’s less complex background.

#### 3.1.2. Performance on Varying Training Sizes

To evaluate the model across different data sizes in the FSL settings, we conducted training with a fixed number of epochs while varying the dataset size. We explored three scenarios using 50, 100, and 200 randomly selected images from the training dataset, with the number of epochs set to 10 for each scenario. Table 2 displays the performance metrics for input image scenarios, including R, mAP in IoU=0.5(mAP@0.5), and mAP in IoU=0.5:0.95(mAP@0.5:0.95).

For dataset $D_{1}$, the model performed well even with only 50 training images, achieving a training R value of 0.926, an mAP@0.5 score of 0.99, and an mAP@0.5:0.95 score of 0.775. On the test set, the performance was slightly lower, with a recall of 0.836, mAP@0.5 of 0.927%, and mAP@0.5:0.95 of 0.724. As the number of training images increased, the test performance consistently improved. With 100 training images, the test recall reached 0.868, and with 200 images, it further increased to 0.897. Correspondingly, the mAP@0.5 improved to 0.953, and the mAP@0.5:0.95 increased to 0.753. These improvements suggest that the model benefits from additional training data and generalizes more effectively with larger datasets.

A similar pattern was observed for the $D_{2}$ dataset. With 50 training images, the model achieved a test R of 0.873, mAP@0.5 of 0.933, and mAP@0.5:0.95 of 0.705. As the training size increases to 100 and then to 200 images, the performance steadily improves. The highest test performance was observed with 200 training images: R of 0.923, mAP@0.5 of 0.963, and mAP@0.5:0.95 of 0.805. Notably, dataset $D_{1}$ exhibited stronger test performance at larger training sizes, particularly in terms of mAP@0.5:0.95.

From Table 2, we observe that the training results for $D_{2}$ are generally lower than those for $D_{1}$, whereas the opposite trend appears on the test set. Although $D_{1}$ is visually simpler, its low diversity and repetitive backgrounds limit the model’s ability to learn strong discriminative features under few-shot conditions. In contrast, $D_{2}$ contains higher visual variability; therefore, a small randomly selected training subset may not represent the overall distribution and may even include more challenging samples than the test set. This can lead to situations in which the model performs better on the test data than on the limited training subset for $D_{2}$.

Overall, the results demonstrate that the model can achieve high accuracy even with a small number of training samples. Its performance improves as more data are provided, showing good scalability and generalization across datasets. These findings highlight the model’s potential for both few-shot learning scenarios and more robust applications, where larger datasets are available. Based on the above analysis, we selected the FSL model trained for 10 epochs on a 50-image set for the ZSL segmentation pipeline.

Although the 200-image setting achieved the highest detection performance, our ZSL segmentation pipeline is designed for operation under strict annotation constraints. The 50-image, 10-epoch configuration provided the best balance between accuracy, annotation efficiency, and model stability, which aligned with the core objectives of few-shot learning. Notably, this setting already produced strong detection performance (mAP@0.5 > 0.92 on both datasets), which was sufficient for generating reliable bounding-box prompts for the SAM2. Although larger training sets improved the metrics, the additional annotation cost contradicted the motivation of the FSL–ZSL framework. Furthermore, the 10-epoch model consistently avoided underfitting (5 epochs) and overfitting (20 epochs on $D_{2}$), yielding stable predictions that are essential for downstream segmentation.

### 3.2. Segmentation Performance

**Figure 6:**
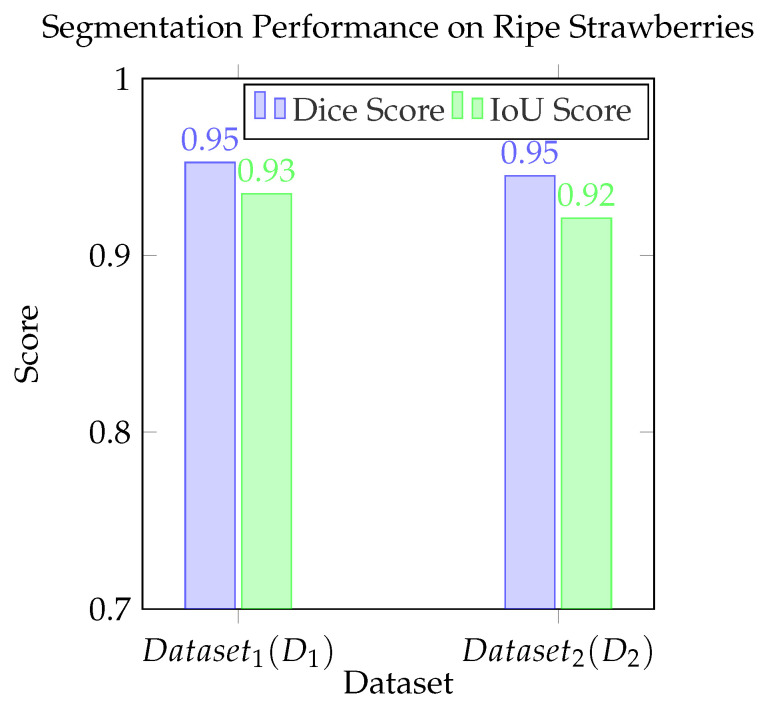
Bar chart comparing mean Dice and IoU scores for ripe strawberry segmentation on $Dataset_{1}\left(D_{1}\right)$ and $Dataset_{2}\left(D_{2}\right)$.

To assess the proposed ZSL segmentation performance, the detection backbone was selected and trained on the 50-image training set with 10 epochs for both the $D_{1}$ and $D_{2}$ datasets. On $D_{1}$, the pipeline achieved a mean DSC of 0.953 and a mean IoU of 0.945 as shown in Figure 6, indicating a high level of segmentation accuracy. On the $D_{2}$ dataset, the model maintained strong performance with a mean DSC of 0.935 and a mean IoU of 0.921.

**Comparative discussion with state-of-the-art (SOTA) methods**: Table 3 presents a comparison of the introduced model with different SOTA methods for strawberry segmentation. We considered both quantitative metrics and qualitative visual examples for the evaluation.

We compared the proposed model with the base model of UNet [25], YOLOv8-seg [26], and YOLOv11-seg [27], as these models are well known for their superior segmentation ability. Furthermore, we explored the impact of the SAM family, such as mobile SAM and SAM along with SAM2, on the segmentation task. For a fair comparison, all models were evaluated using their original configurations and trained under Scenario 1 (Table 1) for 20 epochs for both datasets ($D_{1}$ and $D_{2}$).

**Table 3:** Quantitative comparison against SOTA methods on $D_{1}$ and $D_{2}$. Best results in bold. Inference time is reported per image (ms).

Methods	$D_{1}$		$D_{2}$		Inference Time (ms)
	mIoU	mDice	mIoU	mDice	
UNet [25]	0.855	0.823	0.806	0.818	145.32
YOLOv8-seg [26]	0.855	0.862	0.825	0.845	123.14
YOLOv11-seg [28]	0.889	0.894	0.844	0.871	94.63
YOLOv8 + SAM1	0.930	0.915	0.911	0.890	127.07
YOLOv8 + SAM2	0.938	0.925	0.915	0.909	125.40
YOLOv11 + SAM1	0.939	0.915	0.905	0.903	88.01
YOLOv11 + mobile-SAM	0.9510	0.9305	0.9203	0.9122	86.60
YOLOv11 + SAM2	0.9518	0.9310	0.9374	0.9152	80.02
YOLO-SAM AgriScan (Ours)	**0.9519**	**0.9310**	**0.9509**	**0.9213**	**80.02**

On dataset $D_{1}$, the proposed YOLO-SAM AgriScan achieved notable improvements compared to UNet, raising the mIoU from 0.855 to 0.9519 (+11.3%) and the Dice score from 0.823 to 0.9310 (+13.1%). A similar trend is observed for $D_{2}$, where the mIoU increases from 0.806 to 0.9509 (+14.4%) and the Dice from 0.818 to 0.9213 (+10.3%). Compared with YOLOLv8-seg and YOLOv11-seg, we also observed improved performance of our model. On $D_{1}$, the mIoU improves from 0.889 to 0.9519, increasing mIoU by 7.1%, while the Dice score rises from 0.894 to 0.9310 (+4.1%). On $D_{2}$, the mIoU increases from 0.844 to 0.9509 (+12.7%) and the Dice score from 0.871 to 0.9213 (+5.8%), showing that integrating SAM2 into the proposed architecture substantially refines the segmentation quality beyond detection-driven baselines.

In comparing the proposed approach with models from the SAM family, we focused on the recent YOLOv11-mobile SAM and YOLOv11-SAM models. The YOLOv11-mobile SAM is a lightweight model that demonstrates strong performance, achieving mean intersection over union (mIoU) and Dice coefficients of 0.9510 and 0.9305 on dataset $D_{1}$, and 0.9203 and 0.9122 on dataset $D_{2}$. However, YOLO-SAM AgriScan showed improvements, particularly on $D_{2}$, with an increase of 3.3% in mIoU and 1.0% in the Dice score. This indicates that, while the mobile version effectively balances efficiency and accuracy, the YOLO-SAM AgriScan model achieves superior segmentation precision. We then compared YOLO-SAM AgriScan with its closest variant, YOLOv11-SAM, and observed consistent improvements in performance. The performance on $D_{1}$ is nearly identical for both models; however, on $D_{2}$, the mIoU increases from 0.9374 to 0.9509 (a 1.4% improvement), and the Dice score rises from 0.9152 to 0.9213 (a 0.7% improvement). Although the differences were smaller, these results confirmed that YOLO-SAM AgriScan enhanced boundary precision and segmentation consistency across both datasets.

Table 3 also reports the inference times of YOLO-SAM AgriScan compared with the other methods under the same computational conditions. For fairness, all the images were resized to 640 × 640 pixels. The proposed model achieves a competitive inference time of 80.02 ms per image, outperforming traditional segmentation models such as UNet (145.32 ms) and YOLOv8-seg (123.14 ms) and remaining faster than hybrid baselines such as YOLOv8 + SAM1 (127.07 ms) and YOLOv8 + SAM2 (125.40 ms). This balance between accuracy and efficiency highlights the suitability of YOLO-SAM AgriScan for real-time or near-real-time agricultural applications.

**Figure 7:**
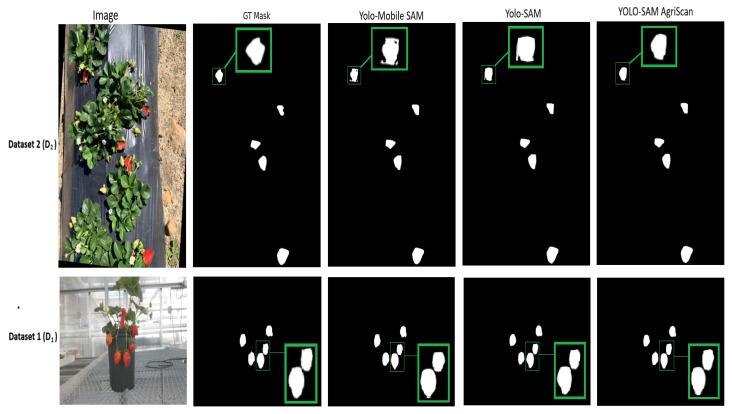
Qualitative assessment on two datasets applying YOLOv11-MobileSAM and YOLOv11-SAM with YOLO-SAM AgriScan.

In Figure 7, we present the qualitative segmentation results for strawberries obtained using the proposed framework. For this assessment, we applied both datasets $D_{1}$ and $D_{2}$. The YOLO-SAM AgriScan was compared with YOLOv11-mobile-SAM and YOLOv11-SAM. Remarkably, we found that the YOLO-SAM AgriScan framework showed enhanced segmentation results that were almost close to the original ground truth. The findings highlight the efficacy of our framework in achieving precise segmentation with potential applications in automated agricultural analysis without manual annotation.

**Figure 8:**
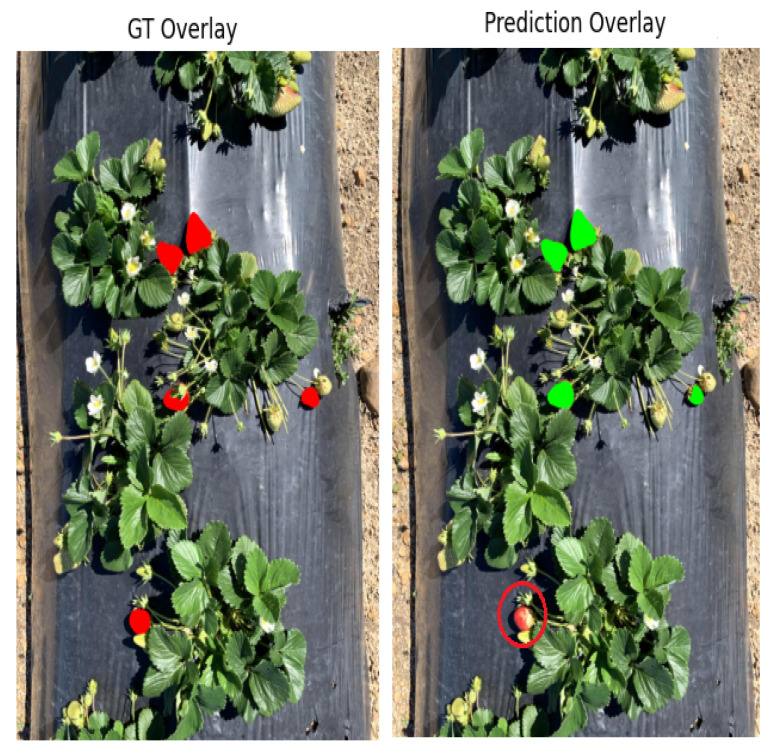
Missed target detection and segmentation case in YOLO-SAM AgriScan.

Figure 8 presents an example from dataset $D_{2}$ in which the proposed method failed to segment the target strawberry (marked with red circle). This failure occurred because the detection model could not localize the strawberries in the first place. The likely cause is the higher level of occlusion and visual complexity within $D_{2}$. In contrast, for our controlled greenhouse dataset ($D_{1}$), we did not observe any missing or absent segmentation.

### 3.3. Potential Applications, Limitations, and Future Works

The proposed YOLO-SAM AgriScan framework has substantial potential for advancing precision agriculture. By integrating FS detection and ZS segmentation, it offers an annotation-efficient approach and reduces the dependence on large, manually labeled datasets, which is a major bottleneck in agricultural AI modeling. This can be deployed for real-time fruit monitoring in greenhouses and open-field environments, enabling automated yield estimation, ripeness assessment, and targeted harvesting. Beyond strawberries, this methodology could be adapted to other fruit crops where visual occlusion remains challenging. The scalability of this approach also positions it for integration into digital twin systems, robotic picking platforms, and intelligent phenotyping pipelines that require consistent instance-level segmentation across diverse environmental conditions.

However, this study is not free from limitations. The segmentation pipeline depends on the accuracy of the YOLOv11-generated Bboxes. As a result, misdetections may propagate to the mask-generation stage. Additionally, model validation was limited primarily to ripe strawberries, and its robustness under varying ripeness stages remains to be comprehensively tested. Future research will focus on enhancing cross-stage generalization by incorporating multiclass maturity detection and spatiotemporal modeling to capture growth dynamics. Further improvements could involve integrating vision–language models for self-adaptive prompting, and 3D multimodal perception combining RGB-D inputs for volumetric segmentation.

## 4. Conclusions

We presented YOLO-SAM AgriScan, a unified and annotation-efficient framework for strawberry detection and segmentation that synergistically combines the strengths of YOLOv11 and SAM2. The proposed approach demonstrated robust performance when training under both limited and full datasets, confirming its scalability and generalization ability. The system achieved consistently high detection precision and recall across varying training epochs and dataset sizes, underscoring its robustness and adaptability to diverse data conditions. The segmentation results further validated the effectiveness of the framework, achieving Dice and IoU scores above 0.82 on both datasets. Compared with earlier fully supervised YOLO + SAM integration methods, our FSL–ZSL strategy reduces annotation requirements while maintaining competitive accuracy. This demonstrated performance highlights the potential of YOLO-SAM AgriScan for real-world agricultural phenotyping applications, including ripeness monitoring, yield estimation, and automated harvesting. However, the accuracy of the framework is constrained by YOLOv11’s detection reliability. Therefore, errors in detection can cascade into segmentation. Furthermore, the segmentation performance was evaluated primarily on ripe strawberries. Future work will enhance cross-stage generalization through multiclass maturity and spatiotemporal modeling, and explore vision–language and RGB-D fusion for volumetric segmentation.

## Figures and Tables

**Table 1:** Experimental training scenarios for YOLOv11-based ripe strawberry detection.

Dataset	Scenario	Split (%)	Training Image	Test Image (20%)	Epochs
$D_{1}$ (300 images)	Scenario 1	80%	240	60	5, 10, 20
	Scenario 2	FS setup	50, 100, 200	60	10
$D_{2}$ (900 images)	Scenario 1	80%	720	180	5, 10, 20
	Scenario 2	FS setup	50, 100, 200	180	10

**Table 2:** Detection performance across different training sizes with 10 training epochs.

Dataset	# Images	Case	Recall	mAP@0.5	mAP@0.5:0.95
$D_{1}$	50	Train	0.926	0.99	0.775
		Test	0.836	0.927	0.724
	100	Train	0.995	0.99	0.772
		Test	0.868	0.942	0.706
	200	Train	0.993	0.995	0.790
		Test	0.897	0.953	0.753
$D_{2}$	50	Train	0.870	0.859	0.649
		Test	0.873	0.933	0.705
	100	Train	0.907	0.925	0.752
		Test	0.907	0.953	0.794
	200	Train	0.884	0.949	0.790
		Test	0.923	0.963	0.805

## Data Availability

The publicly available dataset used in this study is accessible through Roboflow Universe [23]. Additional data generated during the current study are available from the corresponding author upon reasonable request.

## References

[1] Yang Q., Liu L., Zhou J., Rogers M., Jin Z. (2024). Predicting the growth trajectory and yield of greenhouse strawberries based on knowledge-guided computer vision. Comput. Electron. Agric..

[2] Júnior M.R.B., de Almeida Moreira B.R., dos Santos Carreira V., de Brito Filho A.L., Trentin C., de Souza F.L.P., Tedesco D., Setiyono T., Flores J.P., Ampatzidis Y. (2024). Precision agriculture in the United States: A comprehensive meta-review inspiring further research, innovation, and adoption. Comput. Electron. Agric..

[3] Bai Y., Yu J., Yang S., Ning J. (2024). An improved YOLO algorithm for detecting flowers and fruits on strawberry seedlings. Biosyst. Eng..

[4] Bashir A., Ojo M., Zahid A. (2023). Real-time Estimation of Strawberry Maturity Level and Count Using CNN in Controlled Environment Agriculture. Proceedings of the 2023 ASABE Annual International Meeting.

[5] He Z., Khanal S.R., Zhang X., Karkee M., Zhang Q. (2023). Real-time strawberry detection based on improved yolov5s architecture for robotic harvesting in open-field environment. arXiv.

[6] Crespo A., Moncada C., Crespo F., Morocho-Cayamcela M.E. (2025). An efficient strawberry segmentation model based on Mask R-CNN and TensorRT. Artif. Intell. Agric..

[7] Guo Z., Hu X., Zhao B., Wang H., Ma X. (2024). Strawsnake: A real-time strawberry instance segmentation network based on the contour learning approach. Electronics.

[8] Kumar K.S., Safwan K. (2024). Accelerating object detection with yolov4 for real-time applications. arXiv.

[9] Bashir A., Wang Y., Ojo M.O., Zahid A. (2025). A Vision System for Occluded Cutting Point Localization in Robotic Harvesting of Greenhouse Lettuce. IEEE Trans. Agrifood Electron..

[10] de Andrade Porto J.V., Dorsa A.C., de Moraes Weber V.A., de Andrade Porto K.R., Pistori H. (2023). Usage of few-shot learning and meta-learning in agriculture: A literature review. Smart Agric. Technol..

[11] Pourpanah F., Abdar M., Luo Y., Zhou X., Wang R., Lim C.P., Wang X.Z., Wu Q.J. (2022). A review of generalized zero-shot learning methods. IEEE Trans. Pattern Anal. Mach. Intell..

[12] Chen J., Mi R., Wang H., Wu H., Mo J., Guo J., Lai Z., Zhang L., Leung V.C. (2024). A review of few-shot and zero-shot learning for node classification in social networks. IEEE Trans. Comput. Soc. Syst..

[13] Li J., Chen P., Qian S., Liu S., Jia J. (2024). TagCLIP: Improving discrimination ability of zero-shot semantic segmentation. IEEE Trans. Pattern Anal. Mach. Intell..

[14] Ravi N., Gabeur V., Hu Y.T., Hu R., Ryali C., Ma T., Khedr H., Rädle R., Rolland C., Gustafson L. (2024). Sam 2: Segment anything in images and videos. arXiv.

[15] Huang Z., Lee W.S., Takkellapati N.C. (2024). Strawberry Canopy Size Estimation with SAM Guided by YOLOv8 Detection. Proceedings of the 2024 ASABE Annual International Meeting.

[16] Zhao L., Olivier K., Chen L. (2025). An Automated Image Segmentation, Annotation, and Training Framework of Plant Leaves by Joining the SAM and the YOLOv8 Models. Agronomy.

[17] Reddy J., Niu H., Scott J.L.L., Bhandari M., Landivar J.A., Bednarz C.W., Duffield N. (2024). Cotton Yield Prediction via UAV-Based Cotton Boll Image Segmentation Using YOLO Model and Segment Anything Model (SAM). Remote Sens..

[18] Afsar M.M., Bakhshi A.D., Iqbal M.S., Hussain E., Iqbal J. (2024). High-Precision Mango Orchard Mapping Using a Deep Learning Pipeline Leveraging Object Detection and Segmentation. Remote Sens..

[19] Dang Nguyen K., Phung T.H., Cao H.G. (2023). A SAM-based Solution for Hierarchical Panoptic Segmentation of Crops and Weeds Competition. arXiv.

[20] Cheng T., Song L., Ge Y., Liu W., Wang X., Shan Y. Yolo-world: Real-time open-vocabulary object detection. Proceedings of the Proceedings of the IEEE/CVF Conference on Computer Vision and Pattern Recognition.

[21] Yang J., Guo X., Li Y., Marinello F., Ercisli S., Zhang Z. (2022). A survey of few-shot learning in smart agriculture: Developments, applications, and challenges. Plant Methods.

[22] Song Y., Wang T., Cai P., Mondal S.K., Sahoo J.P. (2023). A comprehensive survey of few-shot learning: Evolution, applications, challenges, and opportunities. ACM Comput. Surv..

[23] Objectdetection (2024). Strawberry Seg Dataset. https://universe.roboflow.com/objectdetection-mnlwg/strawberry_seg-zkh1y.

[24] Sekachev B., Manovich N., Zhiltsov M., Zhavoronkov A., Kalinin D., Hoff B., TOsmanov, Kruchinin D., Zankevich A., DmitriySidnev (2020). opencv/cvat: V1.1.0. https://zenodo.org/records/4009388.

[25] Ronneberger O., Fischer P., Brox T. (2015). U-net: Convolutional networks for biomedical image segmentation. Proceedings of the International Conference on Medical image Computing and Computer-Assisted Intervention.

[26] Jocher G., Chaurasia A., Qiu J. (2023). Ultralytics YOLOv8; Version 8.0.0. https://github.com/ultralytics/ultralytics.

[27] Jocher G., Qiu J. (2024). Ultralytics YOLO11; Version 11.0.0. https://github.com/ultralytics/ultralytics.

[28] Khanam R., Hussain M. (2024). Yolov11: An overview of the key architectural enhancements. arXiv.

